# Supplementary material for: Improved Visible Emission from ZnO Nanoparticles Synthesized via the Co-Precipitation Method
Source: Materials (Basel). 2023 Aug 1;16(15):5400. doi: 10.3390/ma16155400 (PMC10420257; doi:10.3390/ma16155400)
Supplement: Supplementary file 1 [file materials-16-05400-s001.zip › materials-2497685-supplementary.pdf]

Supplementary data

for

Improved Visible Emission from ZnO Nanoparticles Synthesized via the Co-Precipitation Method

Alexandra Apostoluk [1], Yao Zhu [1], Pierrick Gautier [2], Audrey Valette [2], Jean-Marie Bluet [1], Thibaut Cornier [2], Bruno Masenelli [1] and Stephane Daniele [2,3,*]

[1] Université de Lyon, INL-INSA Lyon, CNRS, UMR 5270, 69621 Villeurbanne, France; aleksandra.apostoluk@insa-lyon.fr (A.A.)
[2] Université de Lyon, IRCE Lyon, CNRS, UMR 5256, 69626 Villeurbanne, France
[3] C2P2-UMR 5265, ESCPE-Lyon, BP 2077, Univ Lyon, Université Claude Bernard Lyon 1, 69616 Villeurbanne, France
* Correspondence: stephane.daniele@univ-lyon1.fr

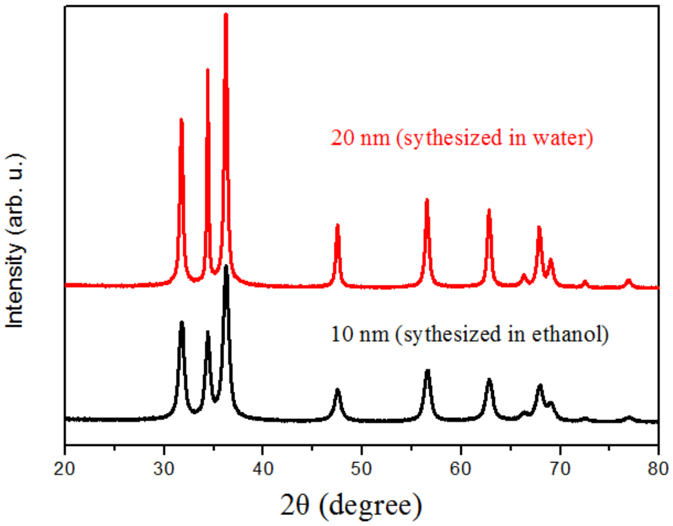

Figure S1: XRD diffractograms of ZnO nanoparticles synthesized by the co-precipitation method of the zinc acetate with KOH (4 KOH/Zn acetate) in ethanol (bottom one) and in water (top one). The curves were moved along the y-axis for the clarity of their presentation.

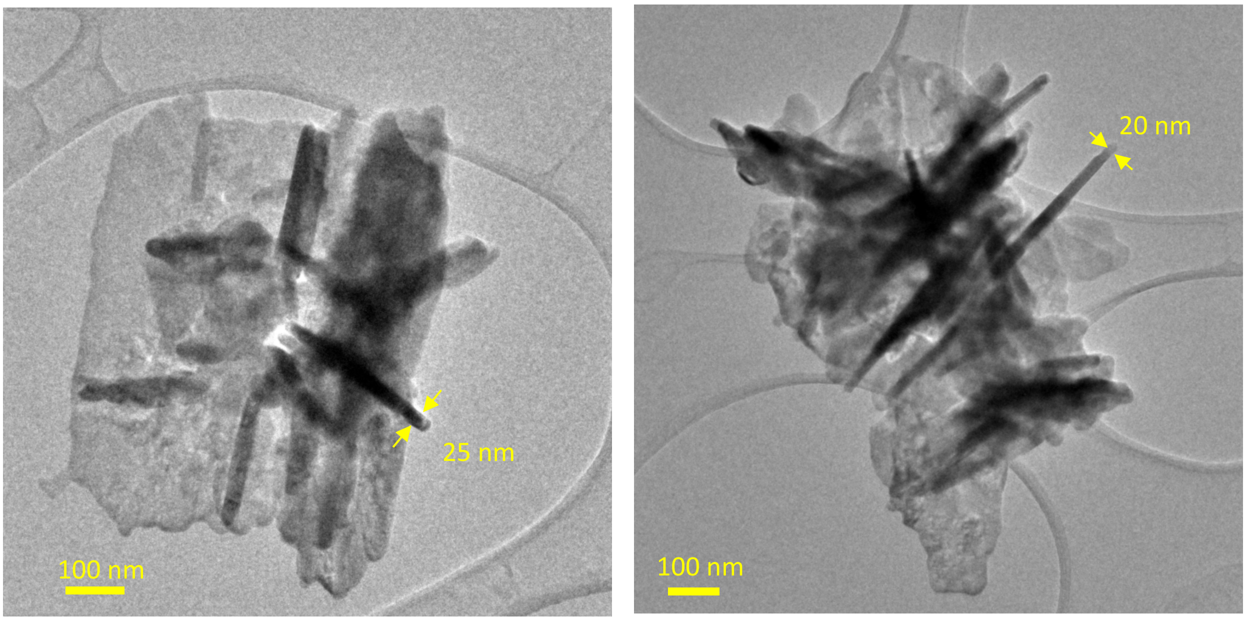

Figure S2: Low magnification TEM images of aggregates of needle-like ZnO structures. The width of the needles is about 20 nm.

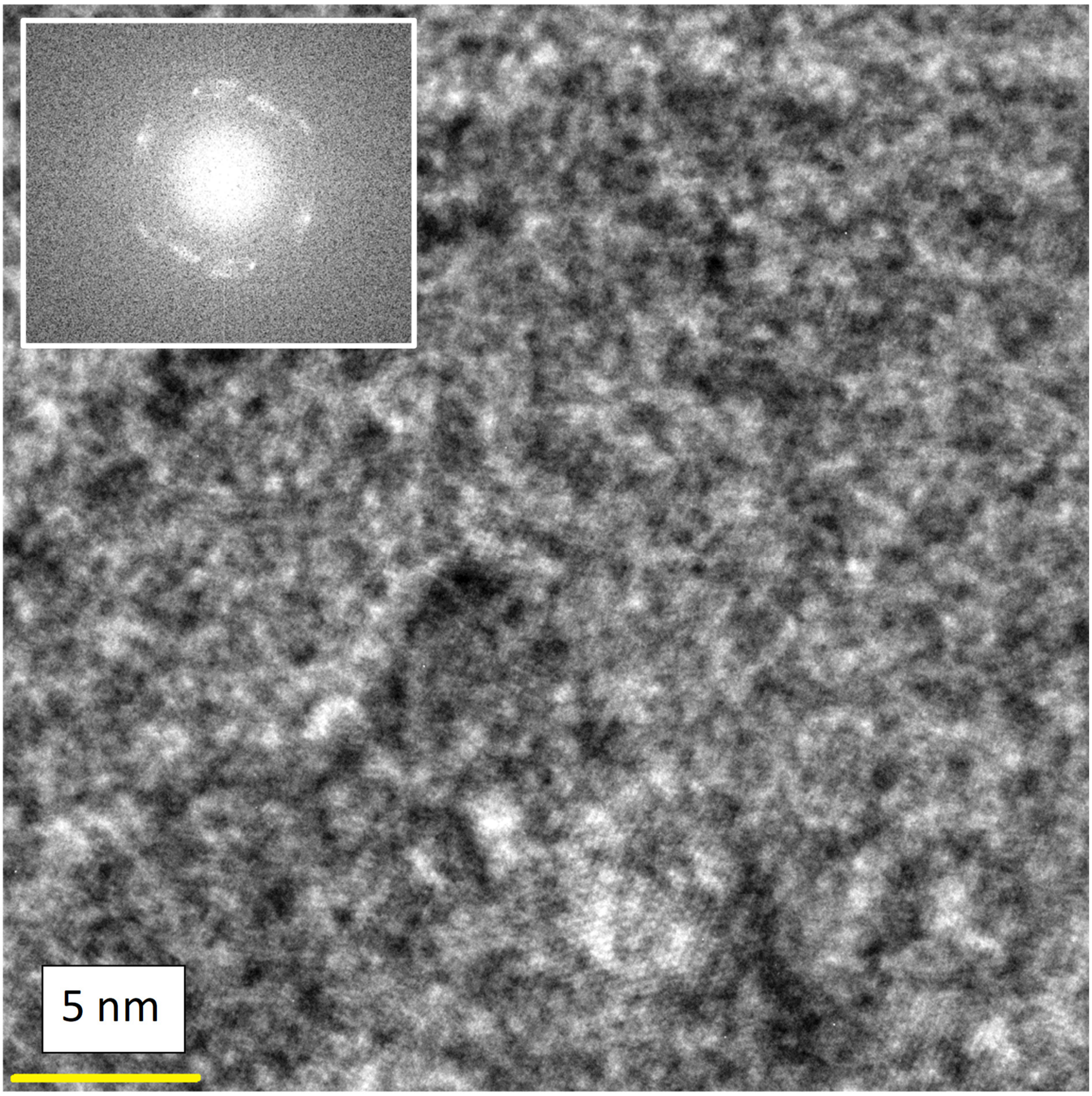

Figure S3: HRTEM image of a needle produced by the co-precipitation of zinc acetate with KOH (4 eq.) in water. The structure is crystallized but is not mono-domain. It exhibits a diffraction pattern (see the Fast Fourier Transform of the image in the inset) which looks like a powder one.

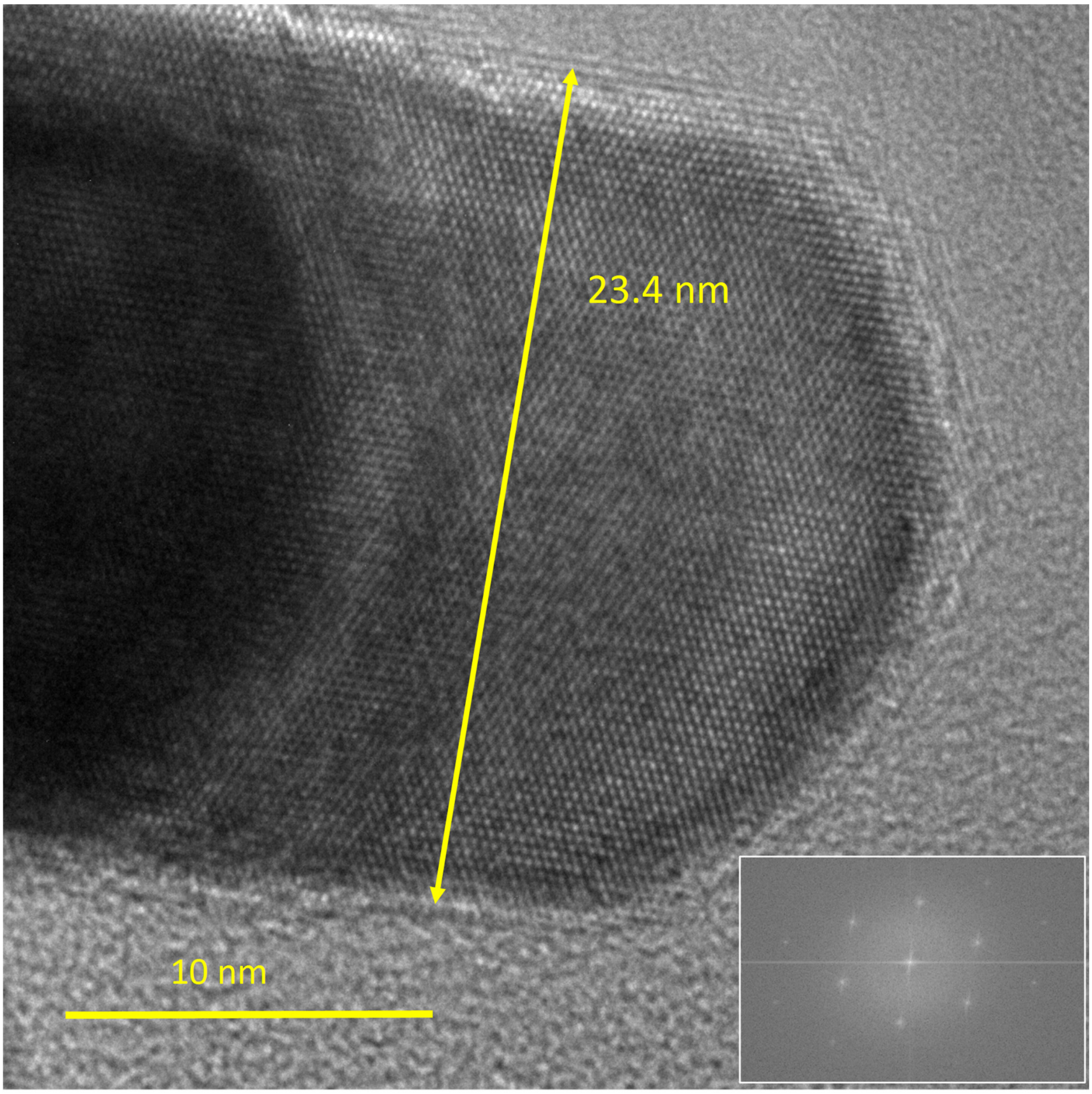

Figure S4: HRTEM image of the tip of a needle produced by the co-precipitation of zinc acetate with KOH (4 eq.) in water. The width of the needle is 23.4 nm. The inset shows the FFT of the image.

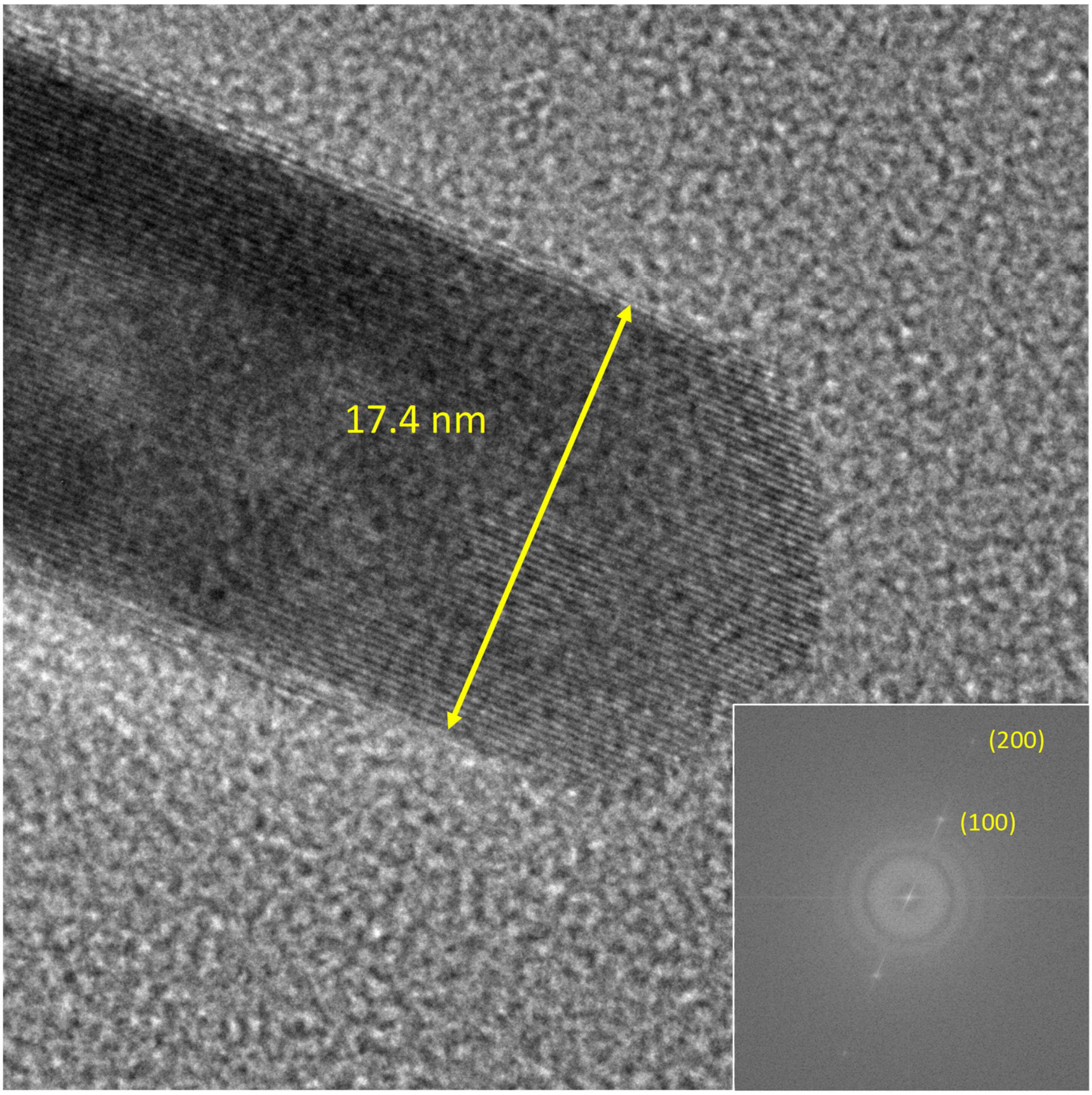

Figure S5: HRTEM image of the tip of a needle produced by the co-precipitation of $Zn(SO_4)_2$ with KOH (4 eq.) in water. The width of the needle is 23.4 nm. The inset shows the FFT of the image.

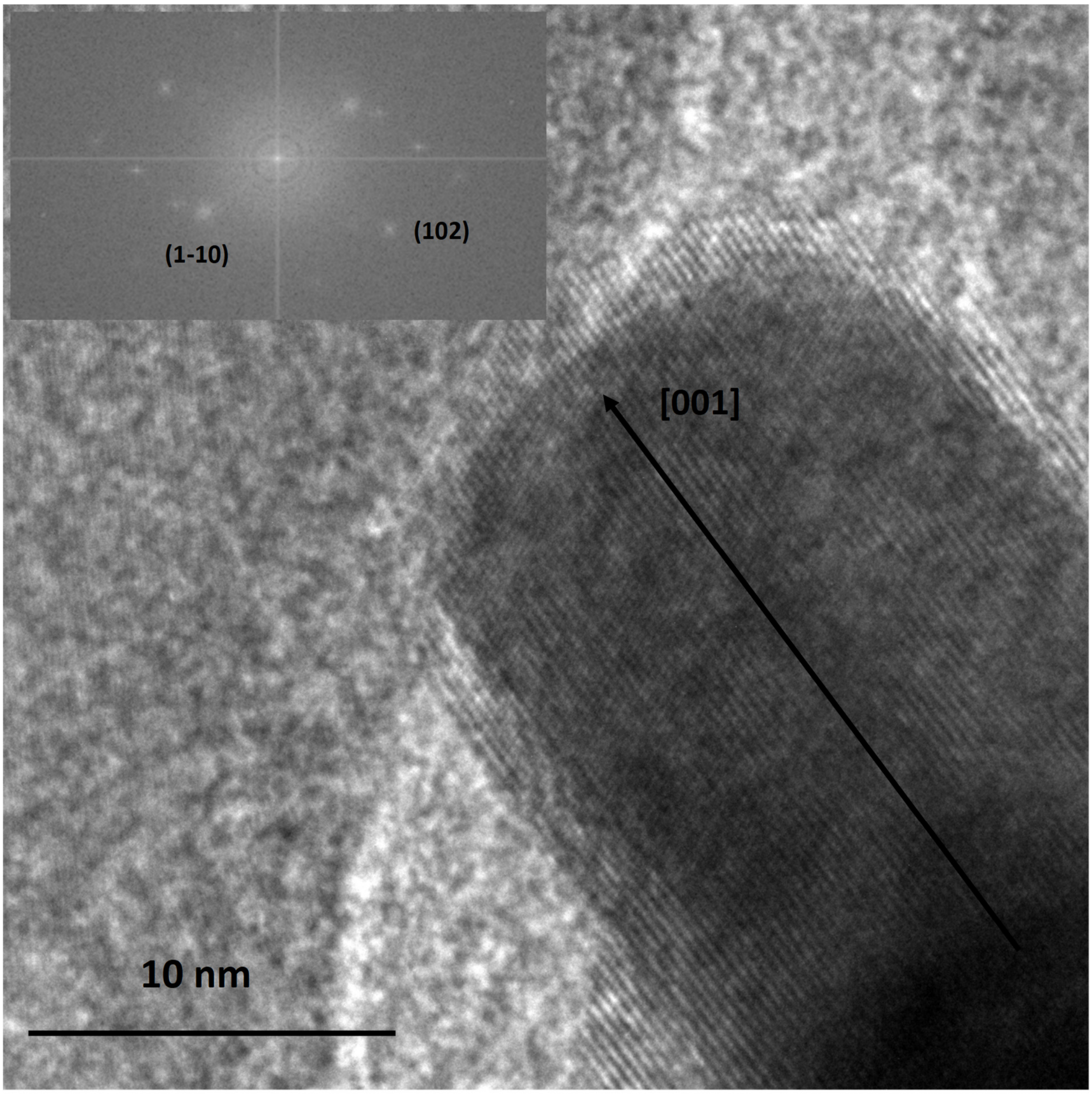

Figure S6: HRTEM image of the tip of a needle produced by the co-precipitation of $Zn(SO_4)_2$ with KOH (4 eq.) in water. The width of the needle is 23.4 nm. The inset shows the FFT of the image.

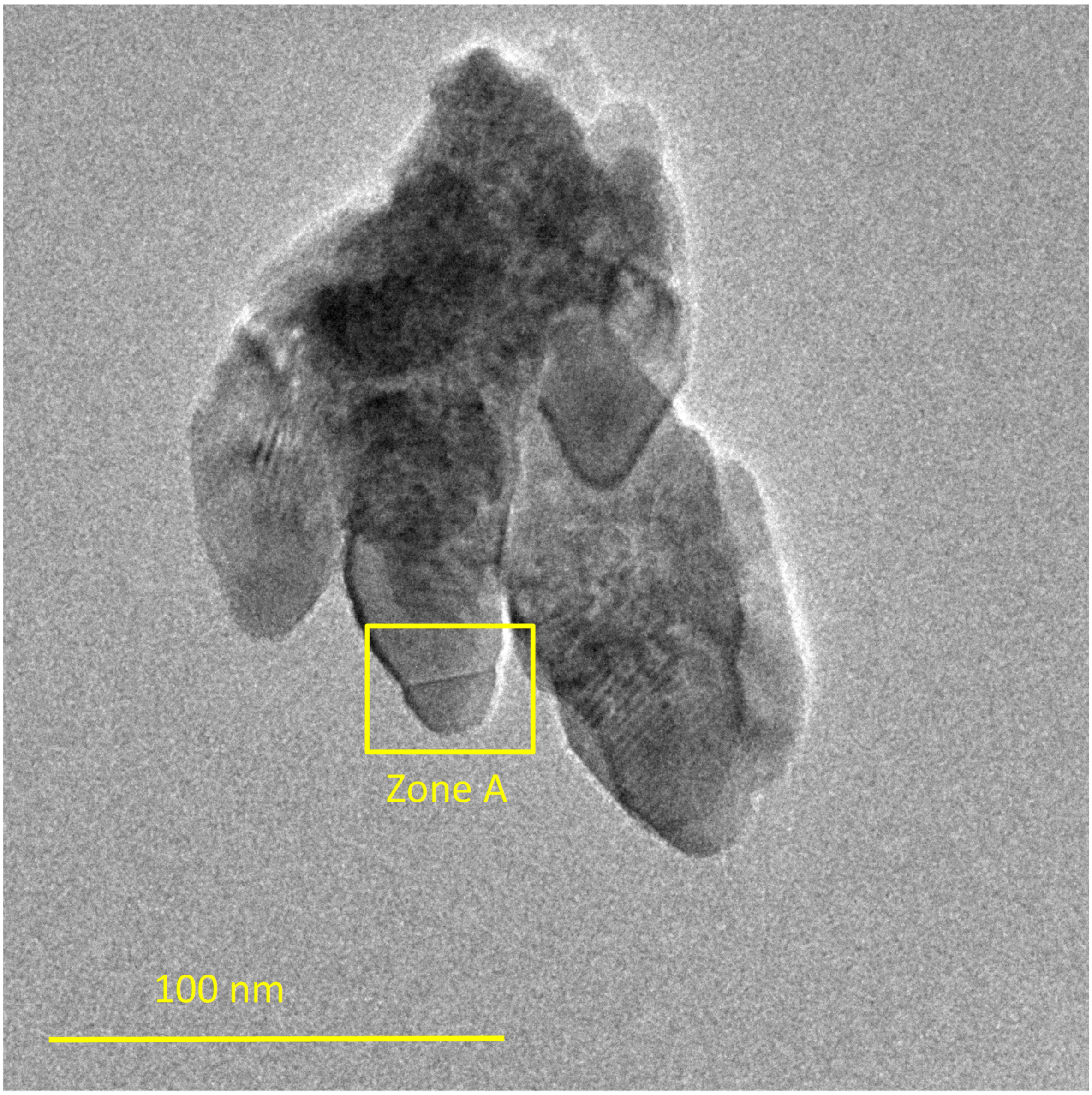

Figure S7: Low magnification TEM images of aggregates of ZnO structures produced by the co-precipitation of zinc acetate with LiOH (4 eq.) in water. Zone A is zoomed in the manuscript (see Figure 6b).
